# Walking boosts your performance in making additions and subtractions

**DOI:** 10.3389/fpsyg.2014.01459

**Published:** 2014-12-19

**Authors:** Filomena Anelli, Luisa Lugli, Giulia Baroni, Anna M. Borghi, Roberto Nicoletti

**Affiliations:** ^1^Department of Psychology, University of BolognaBologna, Italy; ^2^Department of Philosophy and Communication Studies, University of BolognaBologna, Italy; ^3^Institute of Cognitive Sciences and Technologies, Italian National Research CouncilRoma, Italy

**Keywords:** numerical cognition, body motion, embodied cognition, arithmetical calculations, horizontal axis

## Abstract

Previous research demonstrates that the processing of spatial information and numerical magnitude are strictly interwoven. Recent studies also provide converging evidence that number processing is influenced by body movements. In the present study we further investigate this issue by focusing on whether and how motions experienced with the whole body can influence arithmetical calculations. We asked participants to make additions or subtractions while experiencing leftward and rightward motions. Data revealed the emergence of a congruency effect between the orientation inferred by the type of arithmetical calculations and the type of motions experienced along an horizontal axis.

## INTRODUCTION

The first evidence of an influence of number magnitude on space is the so-called spatial-numerical association of response codes (SNARC) effect. During an odd–even classification task on numbers from 1 to 9, responses are faster and more accurate in left space following small numbers (e.g., 2), and in right space following large numbers (e.g., 8), compared to the opposite instructions. This number–response compatibility effect (e.g., Dehaene et al., 1993; for a review, see Wood et al., 2008) suggests a spatial representation of numbers along a continuum on the so-called horizontal mental number line (MNL). In facts, for Western cultures, there is a small-left and large-right order, in which small numbers are positioned on the left and large numbers on the right. The numerical magnitudes along this line seem to be activated by shifts of spatial attention (e.g., Stoianov et al., 2008; Zorzi et al., 2012).

Recently, and more relevant to our aims, spatial biases have been reported even at level of arithmetic operations. For example, McCrink et al. (2007) carried out a psychophysical experiment in which sets of objects were added or subtracted from one another and participants had to judge whether the final number was correct or incorrect. The authors found a systematic bias toward larger values for additions and toward smaller values for subtractions. This mechanism, which subtends the human ability to approximate arithmetics, was defined as Operational Momentum (OM). In addition, a link between space, numbers, and calculations was reported by using different paradigms, for instance: performing constant-speed arm movements (Wiemers et al., 2014), pointing to where the digit would be located on a number line after computing additions or subtractions (Pinhas and Fischer, 2008), or selecting with the mouse the most plausible result of an arithmetic problem at seven possible locations on the screen (Knops et al., 2009). It is worth noting that in the study by Pinhas and Fischer (2008), the authors found a selective rightward bias with addition and leftward bias with subtraction, defined by the authors as spatial OM (i.e., SOM, see also in Pinhas et al., 2014). Furthermore, Pinhas et al. (2014) investigated whether also arithmetic operation signs have spatial connotation by asking participants to decide if a presented sign was a plus or minus and to press a left or right button under two counterbalanced response rules. The authors reported that also arithmetical operation signs can induce spatial associations (i.e., an association between the minus sign with left space and between the plus sign with right space) that may contribute to spatial biases in arithmetic performance (Operation Sign Spatial Association, OSSA effect).

Crucially, the influence of performing movements with the body (i.e., action-related processes) on number processing has received growing attention. For example, random number generation tasks seem to be influenced both by actively and passively experienced movements. More specifically, Loetscher et al. (2008) showed that active head turns to the left and right led participants to generate smaller and larger numbers, respectively, in a random number generation task. Grade et al. (2013) investigated the influence of the passive observation on the random number generation task, and showed that participants generated more small than large numbers after observation of leftward or downward gazes, whereas rightward and upward gazes did not affect the number magnitude.

Furthermore, recent studies have proved the influence of whole body movements, and not only of single body parts, on number generation. For instance, Hartmann et al. (2012b) demonstrated that the passive experience of horizontal and vertical motions (i.e., motions experienced while participants were seated on a moving platform) modulates the number generation task, with a selective facilitation for small numbers when participants were leftward and downward displaced, and a facilitation for large numbers with a rightward and upward displacement.

Of particular relevance is a study by Lugli et al. (2013) that showed a congruency effect when additions and subtractions were executed while moving along the vertical axis. More precisely, the authors asked participants to make additions or subtractions while performing (on-line condition) or after having experienced (off-line condition) an ascending or descending motion through a passive (i.e., taking the elevator) or an active (i.e., taking the stairs) mode. Results showed a congruency effect between the direction of the experienced motion and the orientation related to the type of calculation made. This effect emerged only in the on-line condition and when participants experienced a passive motion. The findings obtained by Lugli et al. (2013) suggested that not only the absolute numerical magnitude, but also the processes leading to the numerical magnitude (i.e., the arithmetic calculations), are connected with the processing of spatial information and are influenced by movements performed with the whole body.

The present study aims to broaden the findings obtained by Lugli and co-authors. In particular, we investigate whether movements executed with the whole body along the horizontal axis (i.e., leftward and rightward motions) can influence the processing of numerical magnitude when calculations had to be performed. To the best of our knowledge, so far only the study by Shaki and Fischer (2014) has investigated how the direction of a lateral turn can influence number processing, by asking participants to generate random numbers while they made lateral turns. Results indicated that participants generated smaller numbers when turning left and larger numbers when turning right, under conditions of both free choice of turn (Experiment 1) and prescribed turn-taking (Experiment 2). Thus, the novelty of the present study consists in testing whether and how leftward and rightward motions, actively performed and experienced with the whole body, influenced arithmetical calculations (i.e., subtractions and additions) instead of the random number generation task.

We hypothesized a congruency effect between the spatial orientation related to the type of calculation performed (i.e., subtractions-leftward orientation and additions-rightward orientation) and the direction of the motion contemporary experienced (i.e., leftward and rightward motions). More specifically, we predicted a facilitation when participants were required to make subtractions while moving toward the left and additions while moving toward the right (congruent condition) with respect to the reverse assignment (i.e., addition-leftward motions and subtractions-rightward motions, incongruent condition).

## MATERIALS AND METHODS

### PARTICIPANTS

Fifty-two undergraduate students from the University of Bologna (33 female, mean age: 20.3 years, SD 3.3) took part in the experiment for course credit. All had a background in humanities, were naïve as to the purpose of the experiment, and gave written informed consent. Four participants who made three or more calculation errors (corresponding to the mean of the errors of all participants plus two standard deviations) were eliminated, so that the final sample consisted of 48 participants.

### ETHICS STATEMENT

The study was approved by the Psychology Department’s ethical committee of the University of Bologna.

### APPARATUS AND STIMULI

Data collection took place in an open space, in a secluded and shaded area of a municipal park. We chose a natural setting for the experiment in order to reproduce, the most closely as possible, everyday processes and actions, such as basic calculations and walking.

Participants were asked to keep subtracting or adding three to a starting number (e.g., 371) for 22 seconds and to say the result of each calculation aloud (e.g., 368, 365, 362, or 374, 377, 380 and so on, for subtractions and additions, respectively, until the 22 seconds were elapsed). We made sure that the starting numbers: (a) were always composed by three digits (e.g., 371; 587); (b) started with two different digits (i.e., 3 or 5, such as 371 or 588).

### PROCEDURE

**FIGURE 1:**
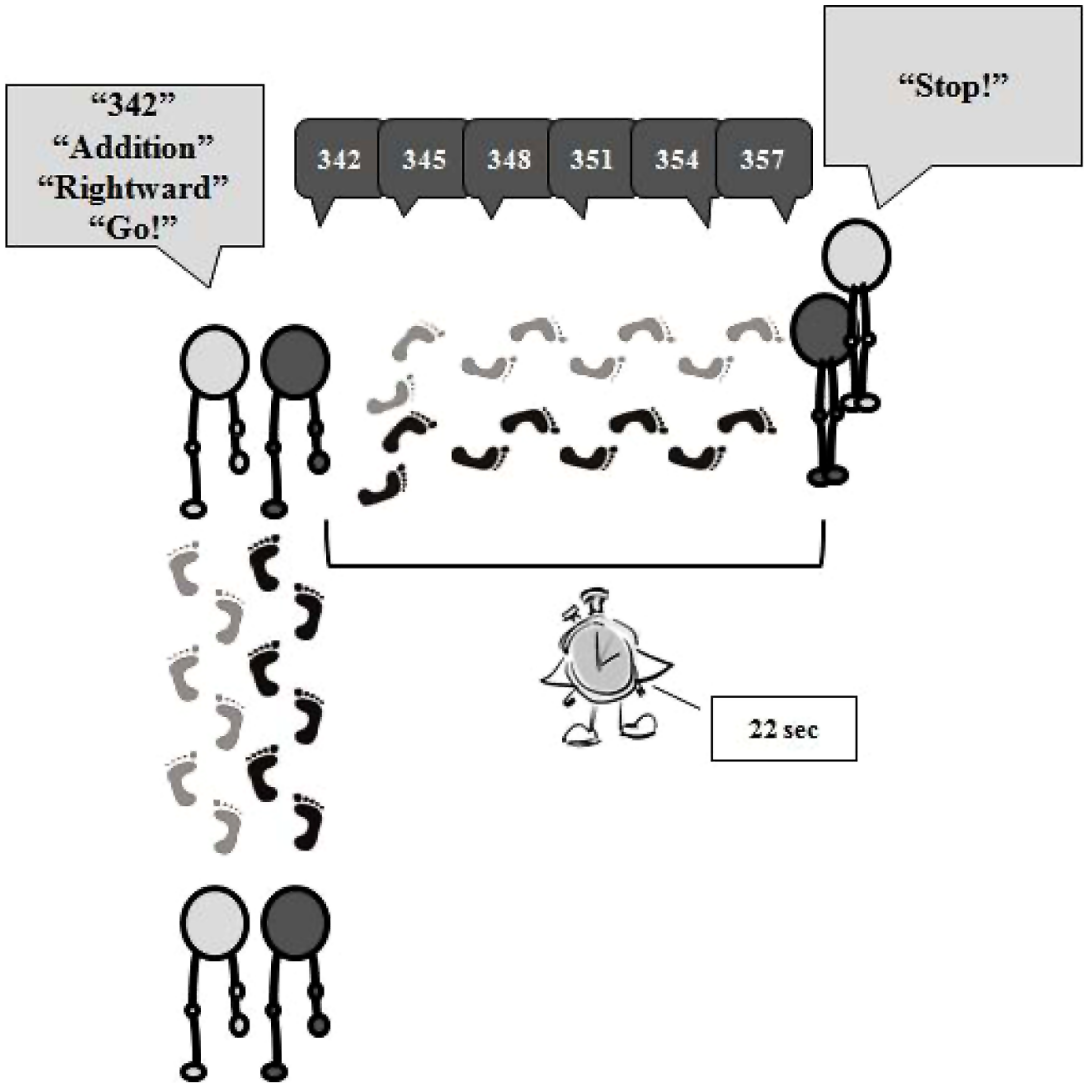
**Schematic representation of the experimental procedure.** At the beginning of each trial, the experimenter and the participant walked close to each other along a straight path (to note, the experimenter randomly changed the position during each trial). Then, the experimenter spoke the starting number aloud, the type of calculation to be executed, the direction of the movement to perform, and then she gave the go signal. Immediately after the go signal, for example, participants turned right and then continued walking for 22 seconds, repeating the starting number and then saying the result of each calculation aloud, until the stop signal.

At the beginning of each trial, the experimenter and the participant walked close to each other along a straight path for 20 seconds. Then, the experimenter spoke the starting number aloud (e.g., 342), informed the participant about the type of calculation to be executed (i.e., subtraction or addition), the direction of the movement to perform (i.e., leftward or rightward turn), and then she gave the go signal. Immediately after the go signal, participants turned left or right and then continued walking for 22 seconds. At the same time, each participant was required to repeat the starting number and then to keep saying the result of each calculation aloud, until the experimenter gave the stop signal (see **Figure 1**). If an error occurred, the trial was repeated giving to the participant a new starting number.

The task was composed by four trials, given by the combination of the two types of calculation (i.e., subtractions and additions) and the two types of motion (i.e., walking leftward and rightward). The type of calculations were always alternated (i.e., a subtraction always followed an addition and vice versa). Throughout the task, the experimenter was in charge of: (a) walking close to the participant (the experimenter randomly changed the position during each trial), so that the number of steps taken was held constant across participants. Hence, the spatial point where each trial started and ended were held constant among participants; (b) recording the start and final numbers of each trial; and (c) keeping track of the calculation errors. No feedback was given during the calculation process and the importance of accuracy over speed was stressed. It is worth noting that two of the authors served as experimenters for this study and that data entry was always double checked before running the analyses. In other words, the experimenter took note of the starting and final number for each trial. At the end of the task, two experimenters computed how many calculations were made for each trial and then entered this value for analyses.

Overall, the experiment lasted about 10/15 min.

## RESULTS

We considered the number of correct calculations as our dependent variable, whereas calculation errors were excluded from analyses.

We hypothesized a congruency effect between the type of motion performed and the type of calculation made. Hence, we divided the trials in congruent (left motions – subtractions; right motions – additions) and incongruent (left motions – additions; right motions – subtractions). Then we averaged the number of correct calculations separately for each group of pairings.

The number of correct calculations were entered into a repeated-measures ANOVA with *Congruency* (congruent vs. incongruent) as the within-subject factor. The magnitude of size effect was expressed by $\eta_{p}^{2}$.

**FIGURE 2:**
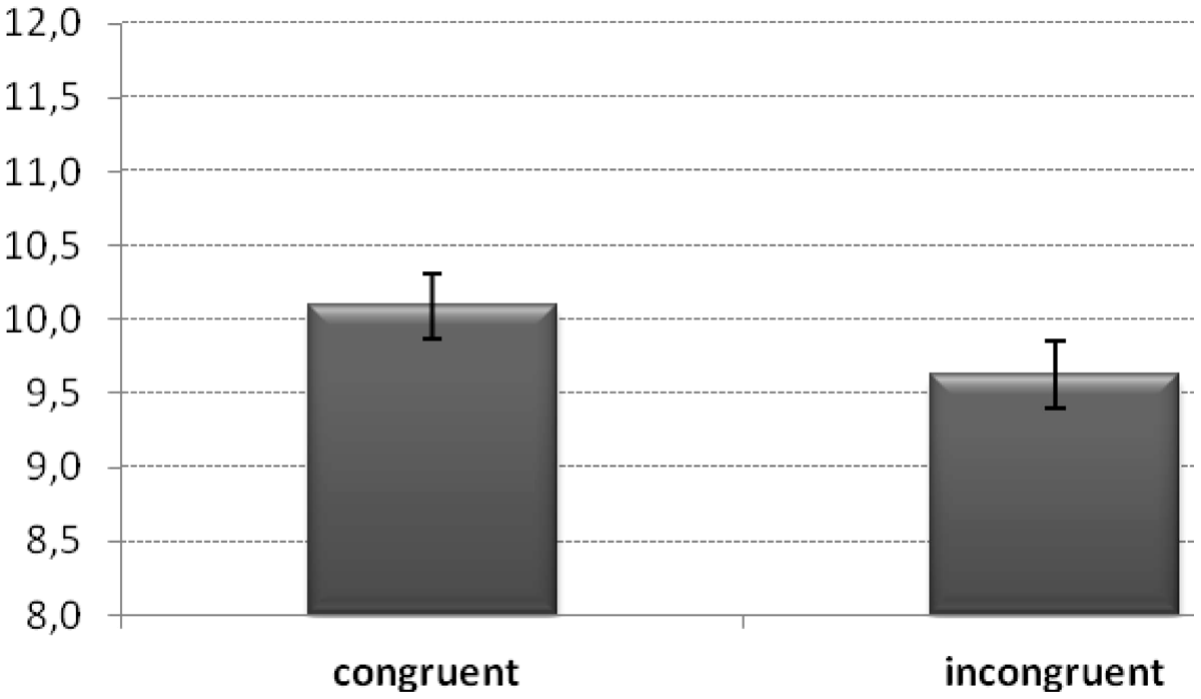
**Number of calculations for congruent (left motions – subtractions; right motions – additions) and incongruent (left motions – additions; right motions – subtractions) pairings.** Values indicate the number of correct calculations and bars are standard error of the mean.

The main effect of *Congruency* [*F*(1,47) = 4.19, *MSE*= 1.26, $\eta_{p}^{2}$ = 0.08, *p* < 0.05] was significant. The number of calculations was higher when participants performed congruent pairings (*M* = 10.1) with respect to incongruent ones (*M* = 9.6; see **Figure 2**; **Table 1**).

**Table 1:** Number of calculations (Mean and SD) as a function of *Congruency* (congruent vs. incongruent) and keeping separate the addition and subtraction.

**Congruency**	**Number of calculations**			
	**Mean**		**SD**	
Congruent	10.1	Additions: 11.1	3.1	Additions: 3.3
		Subtractions: 9.1		Subtractions: 3.3
Incongruent	9.6	Additions: 10.2	3.2	Additions: 3.5
		Subtractions: 9.0		Subtractions: 3.3

We also conducted a congruency analysis separately for additions and subtractions. As far as additions are concerned, the main effect of *Congruency* [*F*(1,47) = 10.79, *MSE*= 1.87, $\eta_{p}^{2}$ = 0.19, *p* < 0.01] was significant. The number of calculations was higher when participants performed congruent pairings (*M* = 11.1) with respect to incongruent ones (*M* = 10.2). As to subtractions, the *Congruency* factor [*F*(1,47) = 0.00, *MSE*= 2.65, $\eta_{p}^{2}$ = 0.00, *p*= 0.95] was not significant, since the number of calculations did not differ between congruent (*M* = 9.1) and incongruent pairings (*M* = 9).

On the whole, results revealed the emergence of a congruency effect between the type of motion performed and the type of calculation made. A facilitation effect was indeed obtained when participants performed calculations in response to congruent pairings (i.e., left motions – subtraction; right motions – additions), instead of incongruent ones (i.e., left motions – additions; right motions – subtractions). The reasons why the congruency effect we found was mainly triggered by additions will be discussed in the further section.

## DISCUSSION

In the present study we explored the link between space and numbers, and in particular whether and how active motions experienced with the whole body influenced the calculation processes. Results showed a facilitation, in terms of higher number of calculations, when additions and subtractions were executed in a congruent pairing (i.e., left motions – subtraction; right motions – additions) rather than in an incongruent one (i.e., left motions – additions; right motions – subtractions). Thus, in line with our hypothesis, data revealed a congruency effect between the direction of the whole body motions and the orientation inferred by the type of calculation processes (i.e., leftward for subtractions and rightward for addictions).

Overall, our data support a bidirectional influence between conceptual and motor activation, and add new evidence to the studies about the influence of the motor processes over semantic ones (i.e., motor-to-semantic effect, see Badets and Pesenti, 2010, 2011; Badets et al., 2012; Ranzini et al., 2012).

The novelty of the present study lies in two points. First, it provides evidence about the influence of active body movements on the calculation processes of additions and subtractions, extending previous findings on the influence of movements on the processing of random numbers generation (e.g., Hartmann et al., 2012b; Shaki and Fischer, 2014). So far, Hartmann et al. (2012a,b) showed that both horizontal and vertical motions, passively experienced with the whole body, could influence numerical cognition (e.g., small numbers were generated more easily during leftward and downward motions, with respect to rightward and upward motions). Furthermore, a recent study by Shaki and Fischer (2014) explored the link between the magnitude of randomly generated numbers and the direction of a lateral turn-taking. The study provides evidence of an association between number magnitudes and lateral turn decisions, since participants generated on average smaller and greater numbers when asked to turn left and right, respectively. Importantly, the amount of steps taken was not correlated to the random number generation task, confirming that the spatial direction parameter (i.e., left or right turning) is more relevant compared to the magnitude of the movement performed (i.e., how far participants walked). Our finding complements and extends previous results revealing that the direction of body motions can influence not only number magnitude in a number generation task, but also the more complex process of calculations that leads to a numerical magnitude.

Second, our evidence concerns the execution of arithmetical operations while moving horizontally in space. The present results are thus in line and broaden the findings obtained by Lugli et al. (2013) in which a facilitation emerged for addition and subtractions while performing upward and downward movements, respectively.

It is worth noting, though, that, differently from Lugli et al. (2013), in the present study we obtained a congruency effect when participants actively experienced the motion. This apparent inconsistency could be due to the fact that the sense of motion related to taking the stairs is more progressive and less direct, with respect to what experienced while walking, when the motion is perceived as faster, requires less effort, and can be probably considered as more automatic and frequently performed in daily life.

Finally, it is worth mentioning another difference between the present work and that of Lugli et al. (2013) as far as the congruency effect is concerned. Keeping separate additions and subtractions, in the present study, and differently from Lugli et al. (2013), the effect seemed to emerge only for additions (see **Table 1**). Two main causes could underlie this result: the differences between the two arithmetic processes and the differences between the two axes on which the two studies focused, i.e., the horizontal (present study) and the vertical axis (Lugli et al.’s study).

On one hand, some evidence spokes in favor of a distinction between additions and subtractions which appear to draw on different strategies. Solving simple addition problems may rely more on declarative memory and less on quantity understanding (e.g., Domahs and Delazer, 2005), whereas subtraction problem solution seems to be more based on direct calculation, given that subtraction is not usually trained in school to the same extent as addition (e.g., Barrouillet et al., 2008). Participants were probably more sensible to external influences, such as the movements they were performing, when making addictions, since those calculations resulted to be overall easier with respect to subtractions (see **Table 1**). On the contrary, while performing subtractions participants might have been more focused on the calculation process with respect to their body movements. One possible further reason of the asymmetry we found between subtractions and additions could be due to the fact that participants walked in an horizontal and facial space. One could indeed speculate that walking in front of me improves addition, while walking backward improves subtraction. On the other hand, a recent study of Wiemers et al. (2014) showed a more reliable motion-arithmetic compatibility effect for the vertical than for the horizontal axis, demonstrating that “mental calculations operate on representations on numerical magnitude that are grounded in a vertical organized mental number space” (ibidem, p.1). Our results are in line with this view, since we found an effect for both addition and subtraction only when the vertical spatial information is involved (Lugli et al., 2013).

The issue addressed in this study can have interesting implications for an embodied and grounded perspective. This recent view of cognition postulates a link between perception and action (Borghi, 2005; Pecher and Zwaan, 2005; Barsalou, 2008), and claims that both abstract and concrete concepts are grounded in perception-action systems (Borghi and Binkofski, 2014). However, so far few evidence revealed that also abstract concepts are based on sensory-motor experiences. Since numbers constitute an example of abstract concepts, studies on numerical cognition are particularly important to fill this gap (e.g., Pecher et al., 2011; Fischer, 2012; Ranzini et al., 2012; Lugli et al., 2013). A recent study by Badets et al. (2014) revealed that, in a random number generation task, observing a leftward or rightward pointing movement lead to a space–number bias only when it had biological kinematics, whereas observing non-biological movements did not. This finding demonstrates the selective influence of the observation of biological movements on the representation of an abstract concept such as number, supporting the embodied view of numerical cognition.

Our study adds to previous evidence in favor of an embodied nature of number processing by showing that numbers representation is influenced by whole body motions (Fischer and Brugger, 2011). Interestingly, recent studies (Fischer et al., 2011; Moeller et al., 2012; Link et al., 2013) also support the claim of an embodied representation of number, by demonstrating the positive effects of training about the spatial representation of number magnitude on children’s number line estimation accuracy. Indeed, the integration of a spatial numerical task with task-specific bodily movements (i.e., indicating the position of a number along a number line on the floor by walking to the estimated location of that number) led to a more pronounced improvement of the mental number line representation.

Hence, the present findings confirm the existence of a connection among numbers, space, and motor processes, by showing the emergence of a congruency effect when subtractions and additions were calculated while moving also along an horizontal axis, and provide further evidence on the reliability of the real-life condition task that the present study and the work by Lugli et al. (2013) proposed.

## AUTHOR CONTRIBUTIONS

Conceived and designed the experiments: Filomena Anelli, Luisa Lugli, Giulia Baroni, Anna M. Borghi, and Roberto Nicoletti. Performed the experiments: Filomena Anelli and Luisa Lugli. Analyzed the data: Filomena Anelli, Luisa Lugli, and Giulia Baroni. Contributed reagents/materials/analysis tools: Filomena Anelli, Luisa Lugli, and Giulia Baroni. Drafted the paper: Filomena Anelli. Provided critical revisions: Luisa Lugli, Giulia Baroni, Anna M. Borghi, and Roberto Nicoletti.

## Conflict of Interest Statement

The authors declare that the research was conducted in the absence of any commercial or financial relationships that could be construed as a potential conflict of interest.
